# Macrophage-Derived Iron-Bound Lipocalin-2 Correlates with Renal Recovery Markers Following Sepsis-Induced Kidney Damage

**DOI:** 10.3390/ijms21207527

**Published:** 2020-10-13

**Authors:** Christina Mertens, Laura Kuchler, Anna Sola, Roser Guiteras, Stephan Grein, Bernhard Brüne, Andreas von Knethen, Michaela Jung

**Affiliations:** 1 Institute of Biochemistry I, Faculty of Medicine, Goethe-University Frankfurt, 60590 Frankfurt am Main, Germany; Christina.Mertens@med.uni-heidelberg.de (C.M.); laurakuchler@gmx.de (L.K.); b.bruene@biochem.uni-frankfurt.de (B.B.); vonknethen@biochem.uni-frankfurt.de (A.v.K.); 2 Department of Experimental Nephrology, IDIBELL, 08908 L’Hospitalet del Llobregat, Barcelona, Spain; asola@idibell.cat (A.S.); rguiteras@idibell.cat (R.G.); 3 Department of Mathematics, Temple University, Philadelphia, PA 19122, USA; grein@temple.edu; 4 Project Group Translational Medicine & Pharmacology TMP, Fraunhofer Institute for Molecular Biology and Applied Ecology IME, 60590 Frankfurt am Main, Germany; 5 Department of Anesthesiology, Intensive Care Medicine and Pain Therapy, University Hospital Frankfurt, Goethe-University Frankfurt, 60590 Frankfurt am Main, Germany

**Keywords:** lipocalin-2, macrophages, renal tubular epithelial cells, iron, CLP

## Abstract

During the course of sepsis in critically ill patients, kidney dysfunction and damage are among the first events of a complex scenario toward multi-organ failure and patient death. Acute kidney injury triggers the release of lipocalin-2 (Lcn-2), which is involved in both renal injury and recovery. Taking into account that Lcn-2 binds and transports iron with high affinity, we aimed at clarifying if Lcn-2 fulfills different biological functions according to its iron-loading status and its cellular source during sepsis-induced kidney failure. We assessed Lcn-2 levels both in serum and in the supernatant of short-term cultured renal macrophages (MΦ) as well as renal tubular epithelial cells (TEC) isolated from either Sham-operated or cecal ligation and puncture (CLP)-treated septic mice. Total kidney iron content was analyzed by Perls’ staining, while Lcn-2-bound iron in the supernatants of short-term cultured cells was determined by atomic absorption spectroscopy. Lcn-2 protein in serum was rapidly up-regulated at 6 h after sepsis induction and subsequently increased up to 48 h. Lcn-2-levels in the supernatant of TEC peaked at 24 h and were low at 48 h with no change in its iron-loading. In contrast, in renal MΦ Lcn-2 was low at 24 h, but increased at 48 h, where it mainly appeared in its iron-bound form. Whereas TEC-secreted, iron-free Lcn-2 was associated with renal injury, increased MΦ-released iron-bound Lcn-2 was linked to renal recovery. Therefore, we hypothesized that both the cellular source of Lcn-2 as well as its iron-load crucially adds to its biological function during sepsis-induced renal injury.

## 1. Introduction

Acute kidney injury (AKI) is a common feature of critically ill patients in intensive care units (ICU), associated with a high mortality and morbidity [1,2]. Kidney damage and renal dysfunction is a very early event during the course of sepsis, diagnosed in about 60% of patients with septic shock [3]. Renal dysfunction increases the risk of not only sepsis-associated mortality, but also the progression toward chronic kidney disease. Hence, early AKI detection is pivotal to prevent both the course of renal dysfunction and to increase patient survival. There are several commonly used biomarkers to assess renal dysfunction, such as serum creatinine (SrCrea) or blood urea nitrogen (BUN). However, these biomarkers appear very late, peaking only 24–48 h after a renal injury insult. Therefore, a panel of novel biomarkers showed promising effects [4]. Among others, lipocalin-2 (Lcn-2) was proposed to serve as an early appearing biomarker for AKI.

Lcn-2, which is also called neutrophil gelatinase-associated lipocalin (NGAL), is a 25-kDa protein of the lipocalin superfamily of carrier proteins [5]. It is known to be rapidly up-regulated during the course of renal injury due to a variety of stimuli, including ischemia/reperfusion [6] or cisplatin-nephrotoxicity [7], for which it was described to serve as a biomarker. However, Lcn-2 also adopts pivotal roles during renal recovery by fostering proliferation and differentiation of renal tubular epithelial cells [8,9,10]. These rather opposing functions might be explained by either the source of Lcn-2 during the early phases of kidney injury versus the later phases of renal recovery or its functional transporting activity. In this regard, we recently found that the biological activity of Lcn-2 depends on its iron-load, whereby iron-loaded Lcn-2 markedly enhanced renal regeneration in a cisplatin-model in vitro [11]. These observations are in line with other studies, proposing that the protective effects of Lcn-2 in renal ischemia/reperfusion injury might be due to its ability to serve as an iron transporter [12,13,14,15]. Moreover, our group described that the role of Lcn-2 in renal regeneration largely depends on the inflammatory micromilieu of the kidney [8,10].

The progression of sepsis is characterized by an immense inflammatory response [16]. Therefore, innate immune responses as well as inflammatory signaling pathways drive the development of a septic shock associated with multi-organ dysfunction, including the pathogenesis of AKI. The systemic inflammation activates a cascade of events associated with stages of sepsis development and consists of various, partially overlapping pathways and redundant mechanisms. With regard to the kidney, we have previously shown that especially the infiltration as well as the presence of macrophages (MΦ) is crucial for renal injury outcome, both during phases of injury as well as for renal regeneration [17]. Hereby, the MΦ phenotype plays a pivotal role. While pro-inflammatory MΦ prevail during the early phases of renal injury, a shift toward anti-inflammatory MΦ is observed during later stages of renal recovery [18]. MΦ are also critical players for the maintenance of both immune as well as tissue homeostasis and the cross-activation and/or inhibition of signaling cascades and other immune cells. Intriguingly, Lcn-2 was described as a potent modulator of MΦ polarization in the context of ischemia/reperfusion injury [8]. Mechanistically, Lcn-2 is induced in MΦ upon their activation with apoptotic cells that accumulate in renal tissue during the development of kidney injury [19,20,21]. The adoptive transfer of Lcn-2-overexpressing MΦ enhanced renal epithelial cell proliferation, an effect that was significantly reduced upon administration of Lcn-2 neutralizing antibodies or the infusion of Lcn-2 knockdown-MΦ [8].

Although the MΦ phenotype is playing a crucial role during the development of sepsis-induced renal injury and Lcn-2 appears as a critical MΦ phenotype determinant, the effect of MΦ-derived Lcn-2 has so far not been addressed in a CLP-induced sepsis model. Moreover, the decisive role of iron and/or Lcn-2-bound iron has not been elucidated in sepsis-induced kidney damage.

## 2. Results

### 2.1. CLP-Induced Sepsis Fosters Kidney Damage at 24 h after CLP and Lcn-2 Protein Appearance in Serum as Early as 6 h after CLP

Even if kidney damage and renal dysfunction is a very early event during the course of sepsis, no reliable biomarkers were identified so far. Since Lcn-2 is a good biomarker for ischemic or nephrotoxic renal damage, we aimed at identifying its role during the course of sepsis.

**Figure 1:**
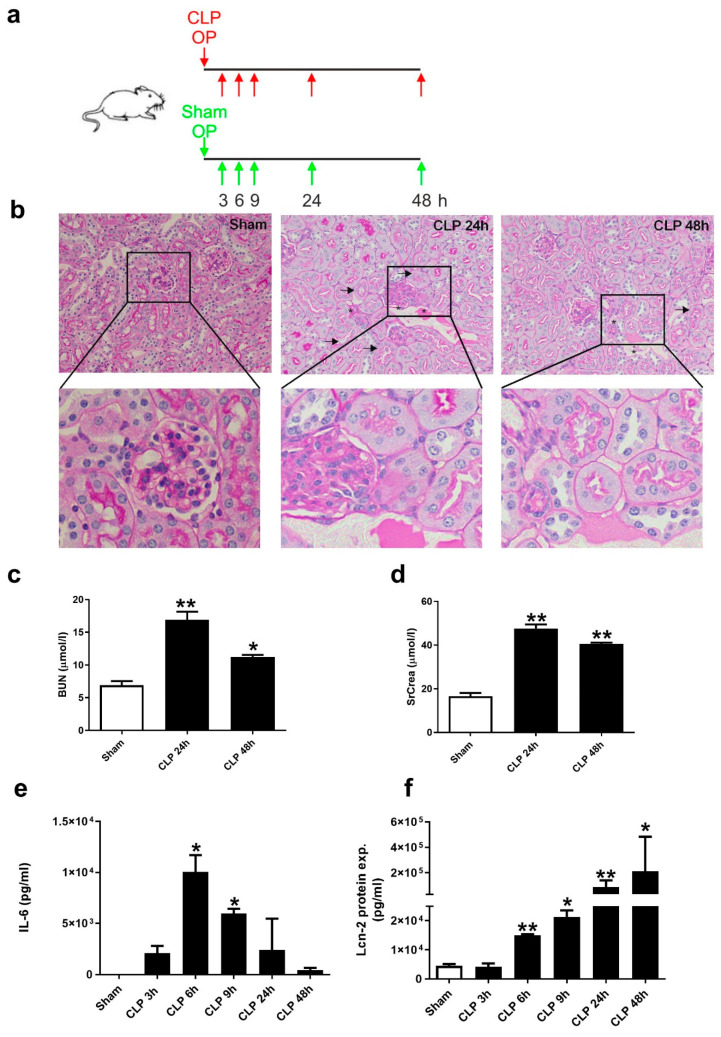
Cecal ligation and puncture (CLP)-induced sepsis promotes kidney damage. (**a**) Schematic overview of the experimental setup of the CLP-model. Sham-operated animals were used as controls. Arrows indicate the time-points of harvesting both blood (3, 6, 9, 24, and 48 h) and kidneys (24 and 48 h) for further processing. (**b**) Histologic analysis of tissue damage applying PAS staining. Pictures are representative of 5 animals in each group. (**c**,d) Analysis of kidney injury markers (**c**) blood urea nitrogen (BUN) (n = 5 animals per group; one-way ANOVA followed by Tukey’s multiple comparison test) and (**d**) SrCrea (n = 5 animals per group; one-way ANOVA followed by Tukey’s multiple comparison test). (**e**) Measurement of IL-6 in serum applying cytometric Bead Array (CBA) (n = 6 animals for 3, 6, and 9 h; n = 5 animals per group for 24 and 48 h; Kruskal-Wallis test followed by Dunn’s post-hoc test). (**f**) Analysis of Lcn-2 protein expression in serum using ELISA (n = 6 animals for 3, 6, and 9 h; n = 5 animals per group for 24 and 48 h; one-way ANOVA followed by Tukey’s multiple comparison test). * *p* < 0.05, ** *p* < 0.01.

For polymicrobial sepsis induction, we used a CLP model by ligating 2/3 of the exposed cecum and puncturing it twice with a 20 G needle (for more details please see Supplementary Figure S1). Sham-operated animals served as controls. The schematic overview in Figure 1a indicates the time-points of harvesting both blood (3, 6, 9, 24, and 48 h) and kidneys (24 h and 48 h) for further processing. Subsequently, we assessed kidney damage by evaluating histology via PAS staining. Kidney morphology showed a moderate deterioration of tissue architecture at 24 h after CLP-treatment. Arrows indicate epithelial cell balloonization as well as detachment and asterisks show epithelial necrosis and interstitial oedema (Figure 1b). Quantification of tissue injury assessment is shown in Supplementary Table S1. Analysis of kidney injury markers serum BUN (Figure 1c) and SrCrea (Figure 1d) showed a significantly increased kidney damage upon 24 h of CLP-induced sepsis, which declined afterwards at 48 h, but remained elevated compared to Sham-conditions. We then measured IL-6 protein amounts in serum of mice and observed a rapid and significant increase at 6 h, which gradually dropped from 9 h to 48 h after CLP (Figure 1e). In contrast, Lcn-2 protein amounts in serum also rapidly increased at 6 h after CLP, but gradually increased during the following time-points (Figure 1f).

### 2.2. Lcn-2 Is Expressed from Different Sources during the Course of Sepsis

**Figure 2:**
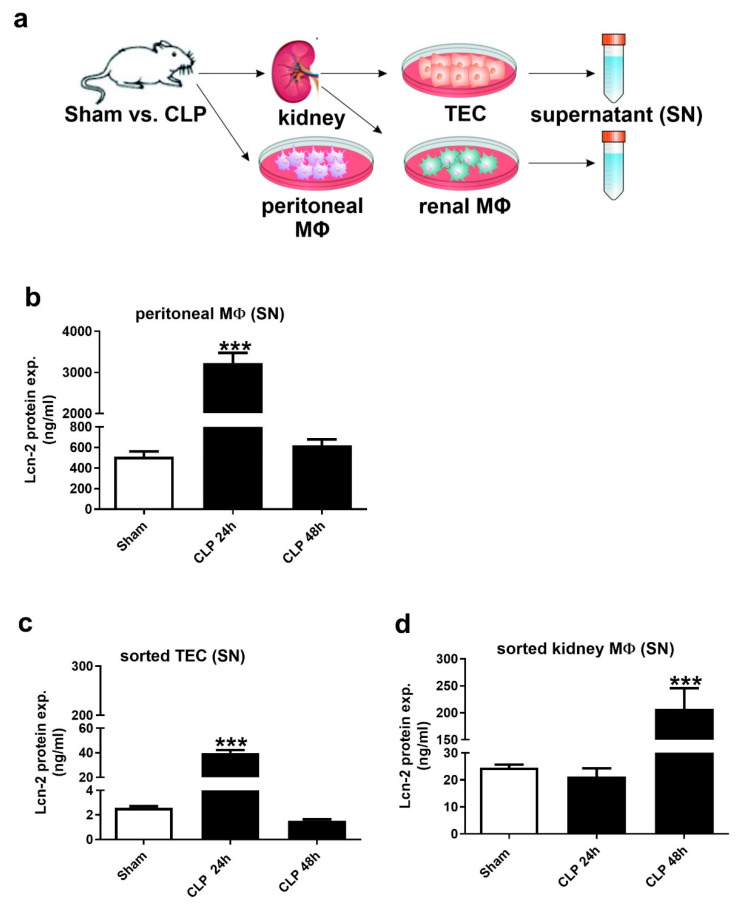
Lcn-2 is expressed from different sources during CLP-induced renal injury progression. (**a**) Schematic representation for the isolation of both macrophages (MΦ) and tubular epithelial cells (TEC). (**b**–**d**) ELISA of Lcn-2 protein expression in the supernatant of short-term cultured and freshly isolated (b) peritoneal MΦ, (c) TEC, and (d) renal MΦ. n = 6 isolated primary peritoneal MΦ, TEC, and renal MΦ per group, *** *p* < 0.001; One-way ANOVA followed by Tukey’s multiple comparison test.

We isolated both peritoneal and kidney MΦ as well as tubular epithelial cells (TEC) (Figure 2a), cultured them for 24 h and analyzed the amount of Lcn-2 protein in the supernatant. The gating strategy for cell sorting is given in Supplementary Figure S2. We found enhanced Lcn-2 secretion in peritoneal MΦ (Figure 2b) as well as TEC (Figure 2c) at 24 h after CLP treatment with a significant drop after 48 h. In contrast, Lcn-2 protein levels in kidney MΦ peaked at 48 h, but remained low at 24 h (Figure 2d).

**Figure 3:**
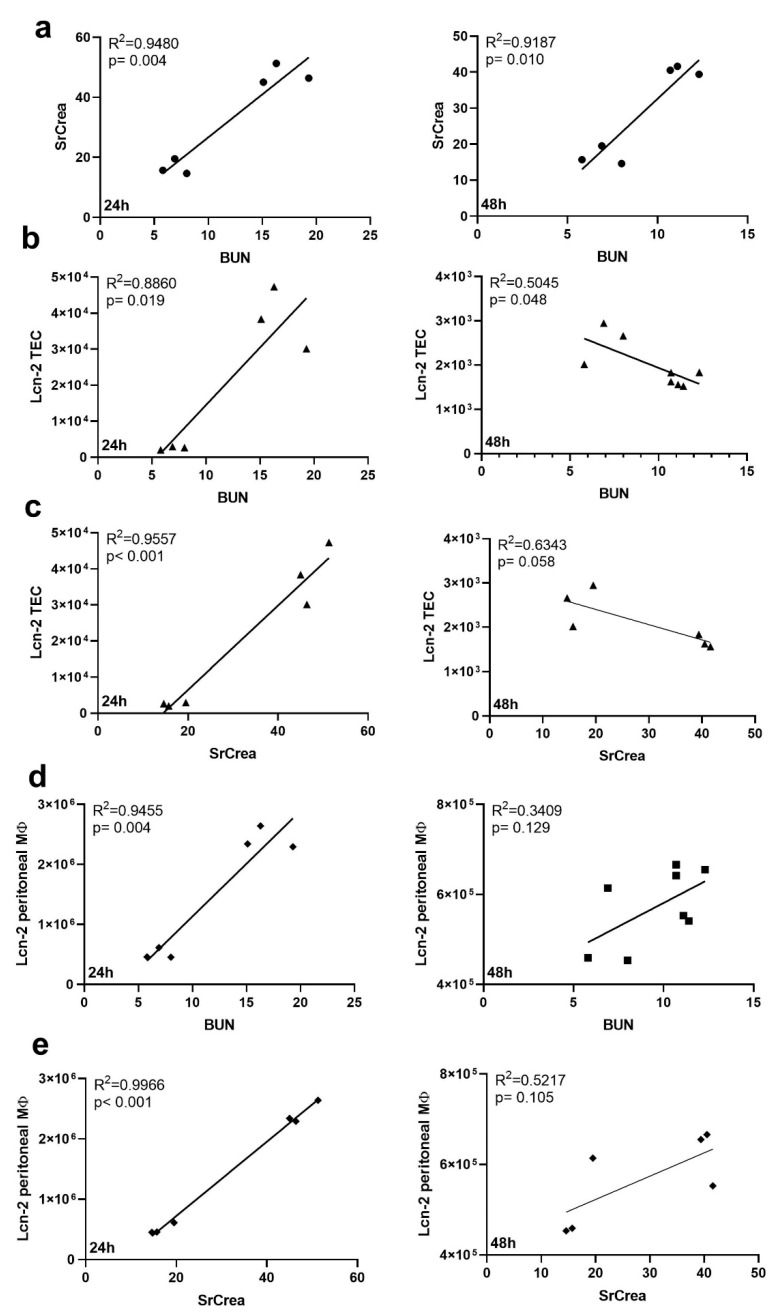
Renal injury markers correlate with Lcn-2 from TEC and peritoneal MΦ during sepsis. (**a**–**e**) Correlation between injury markers BUN (**a**,**b**,**d**) and SrCrea (**c**,**e**) with (**a**) each other or (**b**,**c**) Lcn-2 protein expression in TEC, or (**d**,**e**) with Lcn-2 expression from peritoneal MΦ (separated by time points after CLP treatment, 24 h and 48 h respectively). Both R^2^ and the *p*-value for each correlation are indicated in the individual graph.

We next aimed at identifying whether injury profiles of classical kidney injury markers, i.e., BUN and serum creatinine after CLP-induced sepsis correlated to the release of Lcn-2 as determined in Figure 2. We first confirmed that both injury markers, i.e., BUN and creatinine correlate to each other at 24 h and 48 h (Figure 3a). We then noted that both BUN (Figure 3b,d) and serum creatinine (Figure 3c,e) correlated well to Lcn-2 secreted from TEC (Figure 3b,c) as well as to Lcn-2 levels from peritoneal MΦ (Figure 3d,e) at 24 h after CLP treatment, whereas no significant correlation was observed at 48 h after CLP. However, no correlation of creatinine or BUN to Lcn-2 levels of kidney MΦ (data not shown) was found.

### 2.3. Kidney MΦ Secret Lcn-2-Bound Iron That Correlates with Renal Recovery Markers at 48 h after CLP

**Figure 4:**
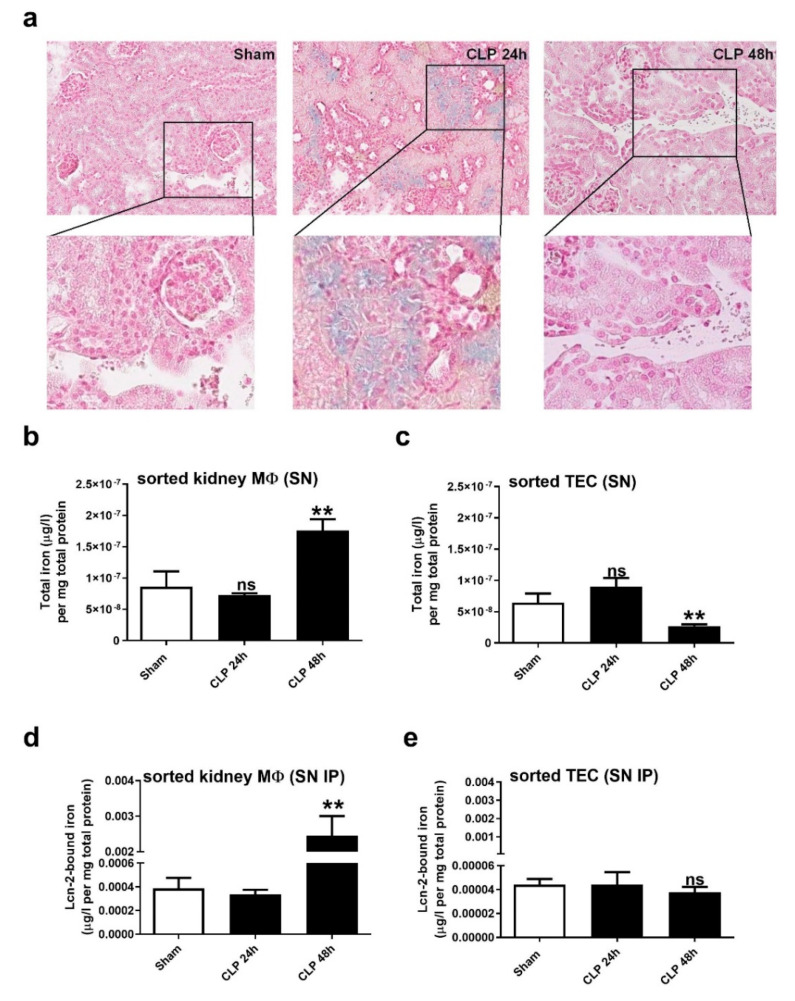
Iron distribution in renal tissue during CLP-induced sepsis. (**a**) Iron accumulation in renal tissue restricted to the lumen of kidney tubules (blue colored areas) at 24 h after CLP detected by Perls’ staining. At the 48 h time-point, only brownish-stained hemosiderin deposits can be observed, which are mainly found in infiltrates. Pictures are representative for 5 animals in each group. (**b**,**c**) Measurements of total iron amounts in the supernatants of short-term cultivated and freshly isolated (**b**) renal MΦ and (**c**) TEC using AAS. (**d**,**e**) Lcn-2 was immunoprecipitated in the supernatants of short-term cultivated and freshly isolated (**d**) renal MΦ and (**e**) TEC, and Lcn-2-bound iron was quantified by AAS. For (**b**–**e**) n = 6 isolated primary peritoneal MΦ, TEC, and renal MΦ per group, ** *p* < 0.01; (**b**–**d**): one-way ANOVA followed by Tukey’s multiple comparison test. (**e**): Kruskal-Wallis test followed by Dunn’s post-hoc test.

Since the complex with iron markedly impacts the biological function of Lcn-2, we first assessed the amount of total iron reservoirs in the kidney after CLP applying Perls’ staining (Figure 4a). We observed nearly no staining in Sham-operated mice, whereas at 24 h after CLP, we noticed a marked increase in positive-stained (blue colored) areas. Interestingly, iron deposits were exclusively found within the lumen of kidney tubules. In contrast, at 48 h after CLP, we detected no iron staining within tubules, but in stromal compartments, which appeared as brownish staining, suggesting to be hemosiderin deposits.

In order to quantify the amount of iron, which is secreted by both TEC and renal MΦ, we measured the iron amount in the supernatant of short-term cultivated TEC and MΦ isolated from either Sham- or CLP-treated mice applying AAS. Interestingly, comparing the total amount of iron in the supernatant of renal MΦ and TEC (Figure 4b,c), we observed an inverse profile for TEC and MΦ. TEC secreted higher levels of iron at 24 h after CLP compared to TEC from Sham-operated mice. In contrast, kidney-isolated MΦ secrete higher levels of iron at the 48 h timepoint. To assess the secretion of Lcn-2-bound iron, we performed immunoprecipitation of Lcn-2 in the supernatant of TEC (Figure 4d) and MΦ (Figure 4e) and then measured the amount of Lcn-2-bound iron via AAS in the immunoprecipitates. Results show no significant changes for Lcn-2-bound iron amounts in TEC, while we determined significantly higher amounts of Lcn-2-bound iron secreted from renal MΦ only at the 48 h timepoint after CLP.

**Figure 5:**
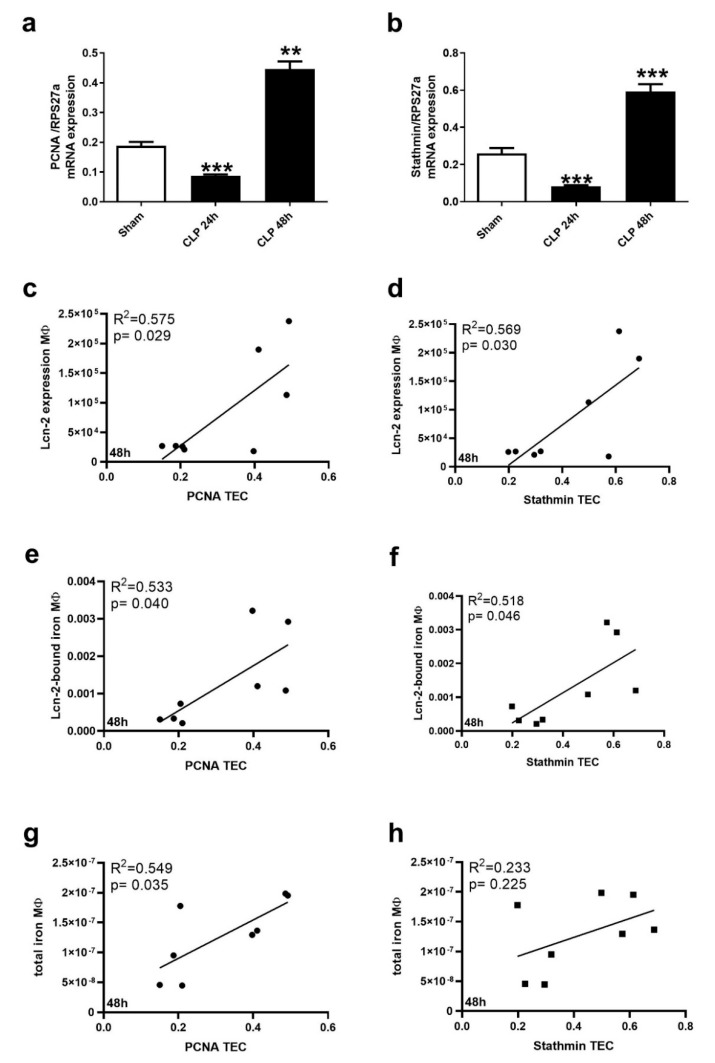
Renal recovery correlates with both total iron as well as Lcn-2-bound iron from renal MΦ. mRNA expression of (**a**) PCNA and (**b**) Stathmin relative to the housekeeping gene RPS27a. n = 5 animals per group. ** *p* < 0.01, *** *p* < 0.001; one-way ANOVA followed by Tukey’s multiple comparison test. (**c**–**h**) Correlation between (**c**,**e**,**g**) PCNA or (**d**,**f**,**h**) Stathmin with either (**c**,**d**) Lcn-2 protein expression, (**e**,**f**) Lcn-2-bound iron, or (**g**,**h**) total iron measured in the supernatant of renal MΦ at 48 h after CLP treatment (all values from this time-point were included; R^2^ and *p*-values are depicted in the individual graph).

In order to evaluate the potential of Lcn-2-bound iron to promote kidney recovery, we measured the regeneration markers PCNA and Stathmin in short-term cultivated TEC from Sham-operated and CLP-treated animals. Both PCNA (Figure 5a) and Stathmin (Figure 5b) showed reduced levels at the 24 h timepoint of CLP, whereas both markers increased at the 48 h timepoint. In order to evaluate if MΦ-derived Lcn-2 or MΦ-secreted iron is associated to these observations, we performed a variety of correlation analyses (Figure 5c–h and Supplementary Figure S3). We did not observe any correlation with recovery markers at the 24 h time-point after CLP (Supplementary Figure S3). However, we found a significant correlation of not only MΦ-derived Lcn-2 with both recovery markers at 48 h after CLP treatment (Figure 5c,d), but also the amount of Lcn-2-bound iron (Figure 5e,f). The total amount of MΦ-secreted iron only correlated to PCNA (Figure 5g), but not to Stathmin levels in TEC (Figure 5h).

### 2.4. Lcn-2 Expression in Patients

**Figure 6:**
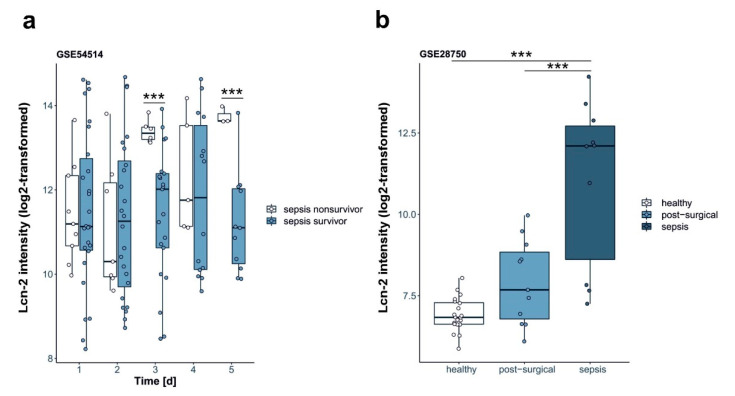
Patient data. Analysis of Lcn-2 expression in the blood of septic patients from publicly available datasets. Data are presented as box- and whisker plots of Lcn-2 expression in the serum. White and gray rectangles represent interquartile range, line in the middle of each rectangle represents the median value. Lines extending from the interquartile range mark the 5th and 95th percentile values, and the individual open circles represent values of each single patient. Analysis of (**a**) Lcn-2 expression in the whole blood transcriptome of survivors and non-survivors of sepsis from Parnell et al. (GSE54514) [22]. (**b**) Lcn-2 expression in PAXgene blood collected for leucocyte RNA isolation and gene expression analysis) from Sutherland et al. (GSE28750) [23]. *** *p* < 0.001.

Supporting the concept that Lcn-2 might correlate with recovery of patients during sepsis we aimed to determine if Lcn-2 expression holds predictive power for septic patients. To this end we analyzed two publicly available data sets [22,23] for the expression of Lcn-2. Parnell et al. (GSE54514) [22] performed gene expression profiling from 2014 to 2018 in whole blood of survivors (n = 26) and non-survivors (n = 9) of sepsis to assess immune suppression compared to healthy controls (n = 18). Samples have been collected daily over a period of five consecutive days. We used this dataset to analyze Lcn-2 expression in the whole blood transcriptome of survivors and non-survivors of sepsis. The expression of Lcn-2 in whole blood positively correlated with survival of septic patients (Figure 6a) on days 3 and 5, whereas no significance was observed at day 1, 2, and 4. Sutherland et al. (GSE28750) [23] distinguished from 2011 to 2019 in a multi-center, prospective clinical trial, patients with sepsis (n = 27) from those who had undergone major open surgery (n = 38) and had clinical outcomes consistent with systemic inflammation. Blood samples were collected within 24 h after surgery. From each participant minimally 5 ml of PAXgene blood were collected for leucocyte RNA isolation and gene expression analyses. In this dataset, we screened for the expression of Lcn2 and compared to healthy controls (n = 20). Lcn-2 expression was positively associated with post-surgical development of sepsis (Figure 6b).

## 3. Discussion

In the present study, we propose that the dual function of Lcn-2 is markedly dependent on its cellular source, which, in turn, determines the outcome of the fine balance between tissue injury and recovery. We present evidence that iron-loaded Lcn-2 is predominantly secreted from tissue MΦ. This correlates with regeneration markers and paves the way toward recovery. In contrast, Lcn-2 in its iron-free form is mostly secreted from injured TEC as well as from peritoneal MΦ, correlating in this latter form with renal injury. Various studies previously identified Lcn-2 as an iron transporter that plays a pivotal role in both acute and chronic kidney disease [24,25,26]. Our study adds to the emerging concept that the iron-load as well as the source of Lcn-2 critically affects its biological function.

Previous studies indicated that Lcn-2 might serve as a good predictor of acute kidney failure in critically ill patients, indicating the need for renal replacement therapy [27,28,29,30]. However, these observations become compromised by the fact that critically ill patients admitted to ICU develop sepsis, which, in turn, leads to the induction of Lcn-2 in a variety of tissues other than the kidney [31,32]. Moreover, the associated systemic inflammatory cascade upon sepsis-induction additionally fosters the expression of Lcn-2 as first-line response of the innate immune system [33,34]. In fact, in the present study, we found high expression levels of Lcn-2 in peritoneal MΦ, suggesting that its secretion, most importantly in its iron-free form, represents a critical part of the innate immune response against invading bacteria. Along these lines, a variety of studies previously addressed the role of Lcn-2 as a bacteriostatic agent [35,36]. Lcn-2 does not bind iron directly but does so with the help of iron-chelating siderophores [26,37,38]. Siderophores are small, high affinity iron-chelating compounds, produced and secreted by bacteria in order to sequester iron [39]. As bacteria acquire most of their iron from the host by synthesizing siderophores, they are able to scavenge and deprive iron from their environment. With regard to the present study results, we speculate that Lcn-2 is produced from peritoneal MΦ upon stimulation of Toll-like receptors by invading bacteria. In turn, secreted Lcn-2 acts as a powerful iron scavenger through its high affinity binding of bacterial siderophores, which is in line with other findings [40]. The biological significance during innate immune responses was underlined using Lcn-2 knockout mice, showing an enhanced susceptibility to a variety of pathogens [36]. Thus, the iron-depriving action of Lcn-2 renders it a potent bacteriostatic agent. We also found high levels of iron-free Lcn-2 secreted from TEC at 24 h after CLP-treatment. These findings concur with the well-established function of Lcn-2 as a biomarker for renal injury development [24,41,42], serving as an acute phase protein, which is rapidly released from affected proximal renal tubules. However, we cannot exclude that Lcn-2 detected in serum might arise from other sources, such as liver or lung. Of note, we observed a significant increase in Lcn-2 expression after 24 h of CLP both in peritoneal MΦ and TEC that drop at 48 h of CLP in these cells. At the 24 h time-point, Lcn-2 levels correlated well with the injury markers. On the contrary, at 48 h after CLP, injury markers still rise, whereas Lcn-2 drops. This time-dependent secretion of established renal injury markers and Lcn-2 as well as their detection might serve as an indicator of early versus late tissue injury phases. Still, more detailed investigations are needed regarding the time window of Lcn-2 expression and secretion, especially during early phases of tissue damage, which are also lacking in our present study. The time of detection of both conventional renal injury markers as well as novel markers such as Lcn-2 may differ significantly until reaching a threshold, where both markers can be detected at the same time. However, this might be exactly the beneficious observation to make so that time points of disease progression could be established, and different treatment regimens might be applicable in order to allow tissue regeneration.

Renal regeneration largely depends not only on the initial damage, but also on local microenvironmental conditions of the tissue [43]. We previously showed that the expression of Lcn-2 in renal MΦ depends on the availability and composition of cytokines within renal tissue [17,21]. Local inflammation also dictates the MΦ phenotype, which, in turn, is crucial during the phase of resolution of inflammation, finally allowing renal recovery [9,21]. Intriguingly, we and others previously described that the MΦ phenotype might also be reflected by the expression and/or repression of iron-regulated genes [44,45,46]. While pro-inflammatory MΦ sequester iron as part of the host defense against invading pathogens, anti-inflammatory-activated MΦ increase their phagocytic activity, efficiently recycling and releasing iron to their microenvironment, which enhances cell proliferation, tissue repair, and regeneration. With regard to Lcn-2, we found that iron-released MΦ also produces and secretes iron-loaded Lcn-2 as part of a complex network of iron-regulated genes in order to promote cellular proliferation [47]. In line, recent work from our group showed that TEC take up MΦ-secreted iron-loaded Lcn-2 in an in vitro cisplatin-induced injury model, whereby proliferation and epithelial cell polarity, as such recovery, was fostered [11].

Although the ability to transport and donate iron results as a pivotal mechanism of Lcn-2 to promote not only cell survival and proliferation, but also to limit tubular damage during acute renal injury [48], the decisive role of the iron-load of Lcn-2 has not been investigated so far in sepsis-induced kidney damage and subsequent recovery progression. Interestingly, results of the present study allow for the assumption that the cellular source of iron-loaded Lcn-2, which is crucial for renal repair [11], are kidney MΦ. We found neither changes in iron-loaded Lcn-2 levels in TEC nor any correlation to TEC-released Lcn-2 with renal regeneration markers PCNA and Stathmin. In contrast, we detected a significantly enhanced production of iron-loaded Lcn-2 from renal MΦ at 48 h after CLP-treatment. In line with our hypothesis, both Lcn-2 production as well as iron-loaded Lcn-2 levels in supernatants of renal MΦ showed a positive association with PCNA and Stathmin levels at 48 h after CLP-treatment. However, further studies are warranted to rule out possible threshold effects regarding the action of iron-loaded Lcn-2 during renal repair and a detailed study is needed to define the window of Lcn-2′s pro-regenerative actions during renal repair mechanisms. Also, mechanistic details are still missing and need to be investigated.

In conclusion, we show that both the cellular source, namely TEC or MΦ, as well as its iron-load define the biological function of Lcn-2 in CLP-induced kidney injury. While enhanced levels of iron-free Lcn-2 from TEC were mainly associated with kidney injury markers at 24 h of CLP-induced kidney injury, increased levels of MΦ-derived, iron-loaded Lcn-2 was associated with recovery markers. However, our study is lacking mechanistic details with regard to activation of Lcn-2-dependent signaling cascades, both at systemic level as well as locally within renal tissue. More studies are required to define how Lcn-2-bound iron is taken up by renal cells and how it is recycled and used within the cell to boost cellular proliferation and survival.

## 4. Materials and Methods

### 4.1. Animals

C57BL/6 wildtype mice were kept in the central research facility of the university hospital Frankfurt. They were housed with water and food ad libitum in rooms with a 12-h light cycle. Organ removal and animal care were performed in accordance with the “Guide for the care and use of laboratory animals” (National Institutes of Health, volume 25, no. 28, revised 1996), EU Directive 86/609 EEC and German Protection of Animals Act.

### 4.2. Sepsis Model—Cecal Ligation and Puncture Model

The cecal ligation and puncture model (CLP) followed the methodology of Rittirsch et al. [49]. Ketamine (Ketavet^®^)/Xylazine (Rompun^®^) 100 mg/200 mg per kg body weight was used for anesthesia. A midline laparotomy incision was done. Two-thirds of the cecum were ligated with an orientation distal to the ileocecal valve. Importantly, the bowel continuity was not disrupted. Following a double puncture using a 20-gauge needle, and applying sufficient pressure to extrude a single droplet of fecal material from each puncture site, the laparotomy was sutured. To avoid dehydration, 1 mL 0.9% NaCl was given to the mice intraperitoneally (i.p.)directly following surgery. Moreover, buprenorphine (Temgesic^®^) 0.5 mg/kg subcutaneously (s. c.) was applied directly after surgery and every 6 h for up to 48 h. For Sham operation, ligation and puncture of cecum was omitted. Kidney damage was determined following CLP. Therefore, in some experiments, blood was taken by heart puncture to isolate serum for determination of the kidney damage markers serum creatinine (SrCrea; abcam, Berlin, Germany) and blood urea nitrogen (BUN; Thermo Fisher Scientific, Dreieich, Germany). Peritoneal MΦ were harvested by injection of 10 mL PBS into the peritoneum and aspiration of the fluid. Cells were kept on ice, washed and cultivated for 3 h in RPMI medium to get rid of non-adhering cells. Before kidney dissection, the organ was flushed with PBS. Afterwards, a part of the kidney was used to prepare a single cell suspension for FACS sorting of tubular epithelial cells (TEC) and renal MΦ. A second part of the kidney was used for hematoxylin/eosin as well as Perls’ staining.

All animal experiments were approved by the State of Hesse animal care and use committee (authorization no. F144/15 and FU/1148).

### 4.3. PAS Stain

For hematoxylin and eosin staining, formalin-fixed and paraffin-embedded tissues were rehydrated, stained using Mayer’s hemalum solution (Merck, Darmstadt, Germany), washed, counter-stained using eosin (Merck), and periodic acid–Schiff and Masson’s trichrome (Merck). Slides were then mounted in entellan (Merck). An Axioskop 40 (Zeiss, Wetzlar, Germany) was used to acquire images.

### 4.4. Cytometric Bead Array (CBA)

IL-6 was measured in the serum of Sham-operated and CLP-treated mice at the indicated time-points. The assay was performed by FACS analysis using the CBA Flex Set for murine IL-6 (BD Biosiences, Heidelberg, Germany). In brief, 25 μL serum fraction and standards were incubated with 25 μL IL-6-coated beads and then labeled with a PE detection reagent. Each incubation procedure was performed for 1 h. Samples were washed with 1 mL FACS flow, centrifuged (200× *g*, 5 min), and resuspended in 300 μL FACS flow for measurement. Samples were acquired with the LSR Fortessa flow cytometer (BD Biosciences, Heidelberg, Germany) and analyzed with BD Biosciences FCAP software.

### 4.5. FACS Sorting

Single cell suspensions of isolated renal tissue from Sham-operated or CLP-treated mice were stained with 7-AAD to detect viable cells and an antibody cocktail containing CD326, CD45, F4/80, and CD11b (all purchased from BD). Cell suspensions were sorted using a FACS Aria (BD) FACS sorter, resulting in CD45-/CD326+ epithelial cells and CD45+/F4/80+/CD11b+ MΦ. The gating strategy is given in Supplementary Figure S1. Cells were then transferred to the cell culture and short-term cultured for 24 h using Dulbecco’s modified Eagle’s medium (Gibco, Dreieich, Germany) for TEC culture and RPMI-1640 medium for rMΦ, each supplemented with penicillin 100 U/mL (Sigma-Aldrich, Taufkirchen, Germany), streptomycin 100 mg/mL (Sigma-Aldrich), and 10% FCS (Capricorn Scientific, Ebersdorfergrund, Germany).

Supernatants were harvested for AAS and cellular lysates were used for RNA isolation. RNA isolation and transcription were performed using the RNeasy Micro Kit (Qiagen, 74004, Hilden, Germany) and Sensiscript RT Kit (Qiagen, 205211) according to the manufacturer’s kit protocols.

### 4.6. Perls’ Staining

Renal tissue slides from Sham- and CLP-treated mice were dewaxed in xylene and rehydrated in a series of alcohol solutions using decreasing concentrations. Perls’ stain was performed using the Iron Stain Kit (Sigma Aldrich, Germany) according to the manufacturer’s protocol. Slides were then washed in distilled water, counterstained with nuclear fast red solution (Sigma Aldrich), rapidly dehydrated, and mounted in entellan (Merck). Pictures were acquired using an Axioskop 40 (Zeiss, Wetzlar, Germany).

### 4.7. Lcn-2 Immunoprecipitation

For immunoprecipitation (IP), supernatants of short-term cultured cells (TEC and renal MΦ) were collected. Dynabeads (Thermo Fisher) were added and incubated overnight at 4 °C in the presence of a specific antibody against Lcn-2 (MAB1857, R&D Systems, Wiesbaden, Germany). Beads were precipitated using the DynaMag-2 magnet (Thermo Fisher) and washed three times with IP buffer. Protein was eluted by addition of 2× loading buffer and incubated at 95 °C for 5 min.

### 4.8. Atomic Absorption Spectrometry

The iron content of TEC and renal MΦ supernatants was determined by graphite furnace atomic absorption spectrometry (AAS). Samples were measured as triplicates with a PinAAcleTM 900 T Atomic Absorption Spectrometer (PerkinElmer, Rodgau, Germany). Slit 0.2 nm and wavelength 248.33 nm were used as spectrometer parameters. A hollow cathode iron lamp (30 mA maximum operating current) was run at 100% maximum current. The calibration solutions (10 µg/L to 90 µg/L) were prepared by adequate dilution of iron standard for AAS (Sigma-Aldrich) stock solution. A pyrolysis temperature of 1400 °C and an atomization temperature of 2100 °C were used.

### 4.9. Quantitative Real-Time PCR (qRT-PCR)

Gene expression profiles were determined by qPCR using the SYBR Green Supermix (Bio-Rad, Munich, Germany) on a CFX-Connect real-time-PCR detection system (Bio-Rad). Results were quantified using the Bio-Rad CFX-Manager (Bio-Rad, version 3) with 18S mRNA expression as housekeeping control. All primers were purchased from Biomers (Ulm, Germany):

PCNA: Fw: 5-AATGGGGTGAAGTTTTCTGC-3, Rv: 5-CAGTGGAGTGGCTTTTGTGA-3

Stathmin: Fw: 5-CTTGCGAGAGAAGGACAAGC-3, Rv: 5-CGGTCCTACATCGGCTTCTA-3

RPS27a: Fw: 5-GACCCTTACGGGGAAAACCAT-3, Rv: 5-AGACAAAGTCCGGCCATCTTC-3

### 4.10. Lcn-2 ELISA

Serum was collected from Sham-operated or CLP-treated mice at the indicated timepoints. A volume of 100 µL of each sample was applied to an ELISA well-plate previously covered with the anti-Lcn-2 (MAB1857, R & D) and blocked for 1 h. After sample incubation, the detection anti-Lcn-2 antibody was added. HRP-conjugated avidin (Invitrogen, Dreieich, Germany) was incubated for 1 h, the color reagent (OPD tablets; Dako, Jena, Germany) was added, and color development was assessed.

### 4.11. GSE Files

Correlation between Lcn-2 expression in the blood of septic patients and illness was carried out. Given data were assessed with gene expression profiles from accessible microarray data sets [22,23]. To this end, we used the following studies from Sutherland et al. (GSE28750) 20, and Parnell et al. (GSE54514) 21. Statistical analysis and graphing of the provided data was conducted with GNU R 3.6.3 for Windows.

To test for statistical significance the Welch two-sample t-test was invoked for GSE 54514. For analysis of GSE 28,750 one-way ANOVA followed by post-hoc Tukey’s HSD test for multiple comparisons was applied.

### 4.12. Statistical Analyses

Statistical analyses were performed applying GraphPad Prism^®^ 8 software (GraphPad Software, San Diego, CA, USA). The distribution of variables was tested for normality using the Kolmogorov-Smirnov test. Accordingly, statistical significance was calculated using one-way ANOVA followed by Tukey’s multiple comparison test or Kruskal-Wallis test followed by Dunn’s post-hoc test, where applicable. Significance of correlations was determined by Spearman’s test including all investigated groups. *p*-values ≤ 0.05 were assumed as statistically significant.

## Supplementary Materials

The following are available online at https://www.mdpi.com/1422-0067/21/20/7527/s1, **Figure S1**: Cecal puncture and ligation model. For polymicrobial sepsis induction, we used the following CLP model: for CLP surgery, mice were anesthetized, and the abdominal cavity is opened via a midline laparotomy incision of about 3 cm in an aseptic fashion (**A**). The cecum was exposed (**B**) and 2/3 of the cecum is ligated distal to the ileocecal valve, taking care to maintain bowel continuity. The ligated cecum was punctured “through-and-through” with a 20-gauge needle (**C**). Next, sufficient pressure was applied to the cecum to extrude a single droplet of fecal material from each puncture site. The abdomen is closed in two layers (**D**), and mice are resuscitated with 1 ml of 0.9% NaCl. Mice subjected to sham laparotomy (Sham) underwent the same procedure without ligation and puncture. **Figure S2**: Gating strategy for cell sorting. Single cell suspensions of isolated renal tissue from Sham-operated or CLP-treated mice were stained with 7-AAD to detect viable cells and an antibody cocktail containing CD326, CD45, F4/80, and CD11b. Cell suspensions were sorted using a FACS Aria (BD) FACS sorter, resulting in CD45-/CD326+ epithelial cells and CD45+/F4/80+/CD11b+ MΦ. **Figure S3**: Correlation to renal recovery markers at 24 h after CLP treatment. (**a**–**f**) Correlation between (**a**,**c**,**e**) PCNA or (**b**,**d**,**f**) Stathmin expression in TEC with either (**a**,**b**) Lcn-2 protein expression in renal MΦ, (**c**,**d**) Lcn-2-bound iron secreted from renal MΦ, or (**e**,**f**) total iron measured in the supernatant of renal MΦ (all values from the 24 h timepoint after CLP were included; R^2^ and p-values are depicted in the individual graph). **Table S1**: Histological scoring. Kidney damage was assessed by evaluating histology via PAS staining. Poor (+), moderate (++), and severe (+++) scoring was applied and kidney sections were analyzed by screening for tubular detachment, tubular dilatation, tubular vacuoles, inflammatory infiltrate, and glomerular sclerosis. Five independent mice were analyzed from Sham- and CLP-treated animals (24 h and 48 h).

## Abbreviations

AAS	atomic absorption spectrometry
AKI	acute kidney injury
BUN	blood urea nitrogen
CLP	cecal ligation and puncture
ICU	intensive care unit
Lcn-2	lipocalin-2
TEC	tubular epithelial cells
MΦ	macrophages
PCNA	proliferating nuclear cell antigen
SrCrea	serum creatinine
TEC	tubular epithelial cells
wt	wildtype
