# Supplementary material for: Macrophage-Derived Iron-Bound Lipocalin-2 Correlates with Renal Recovery Markers Following Sepsis-Induced Kidney Damage
Source: Int J Mol Sci. 2020 Oct 13;21(20):7527. doi: 10.3390/ijms21207527 (PMC7589935; doi:10.3390/ijms21207527)
Supplement: Supplementary file 1 [file ijms-21-07527-s001.pdf]

International Journal of
Molecular Sciences

MDPI

Article

Supplemental Materials: Macrophage-derived iron-bound lipocalin-2 correlates with renal recovery markers following sepsis-induced kidney damage

Christina Mertens[1#], Laura Kuchler[1#], Anna Sola[2], Roser Guiteras[2], Stephan Grein[3], Bernhard Brüne[1,4], Andreas von Knethen[1,4,5], Michaela Jung[1*]

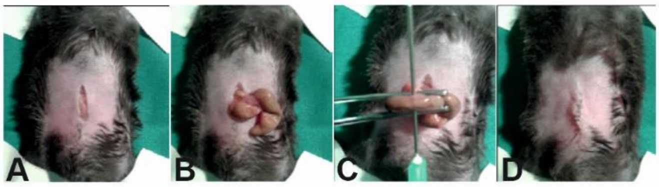

Supplemental Figure S1: Cecal puncture and ligation model

For polymicrobial sepsis induction, we used the following CLP model: For CLP surgery, mice were anesthetized, and the abdominal cavity is opened via a midline laparotomy incision of about 3 cm in an aseptic fashion (A). The cecum was exposed (B) and 2/3 of the cecum is ligated distal to the ileocecal valve, taking care to maintain bowel continuity. The ligated cecum was punctured 'through-and-through' with a 20-gauge needle (C). Next, sufficient pressure was applied to the cecum to extrude a single droplet of fecal material from each puncture site. The abdomen is closed in two layers (D), and mice are resuscitated with 1 ml of 0.9% NaCl. Mice subjected to sham laparotomy (Sham) underwent the same procedure without ligation and puncture.

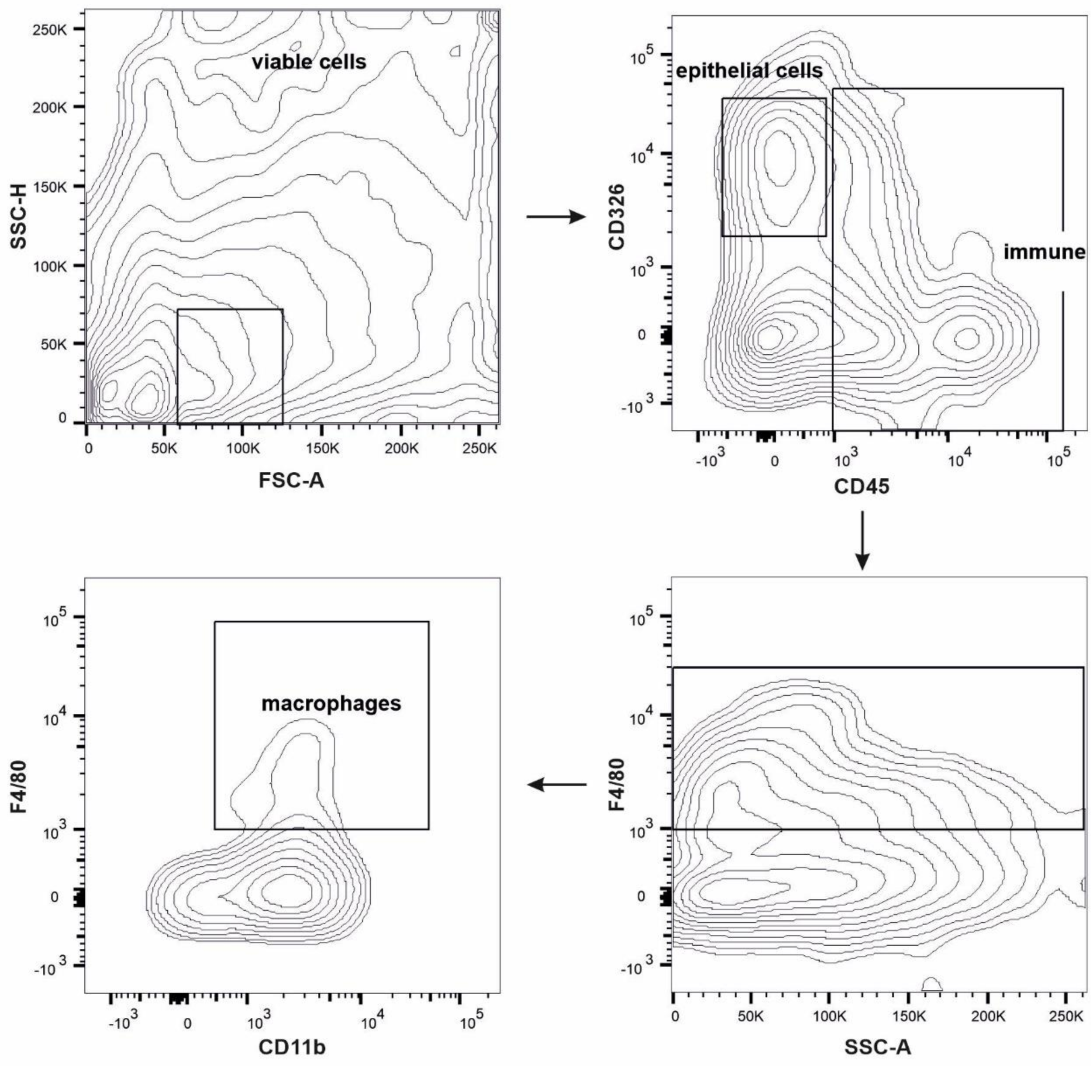

Supplemental Figure S2: Gating strategy for cell sorting

Single cell suspensions of isolated renal tissue from Sham-operated or CLP-treated mice were stained with 7-AAD to detect viable cells and an antibody cocktail containing CD326, CD45, F4/80, and CD11b. Cell suspensions were sorted using a FACS Aria (BD) FACS sorter, resulting in CD45-/CD326+ epithelial cells and CD45+/F4/80+/CD11b+ MΦ.

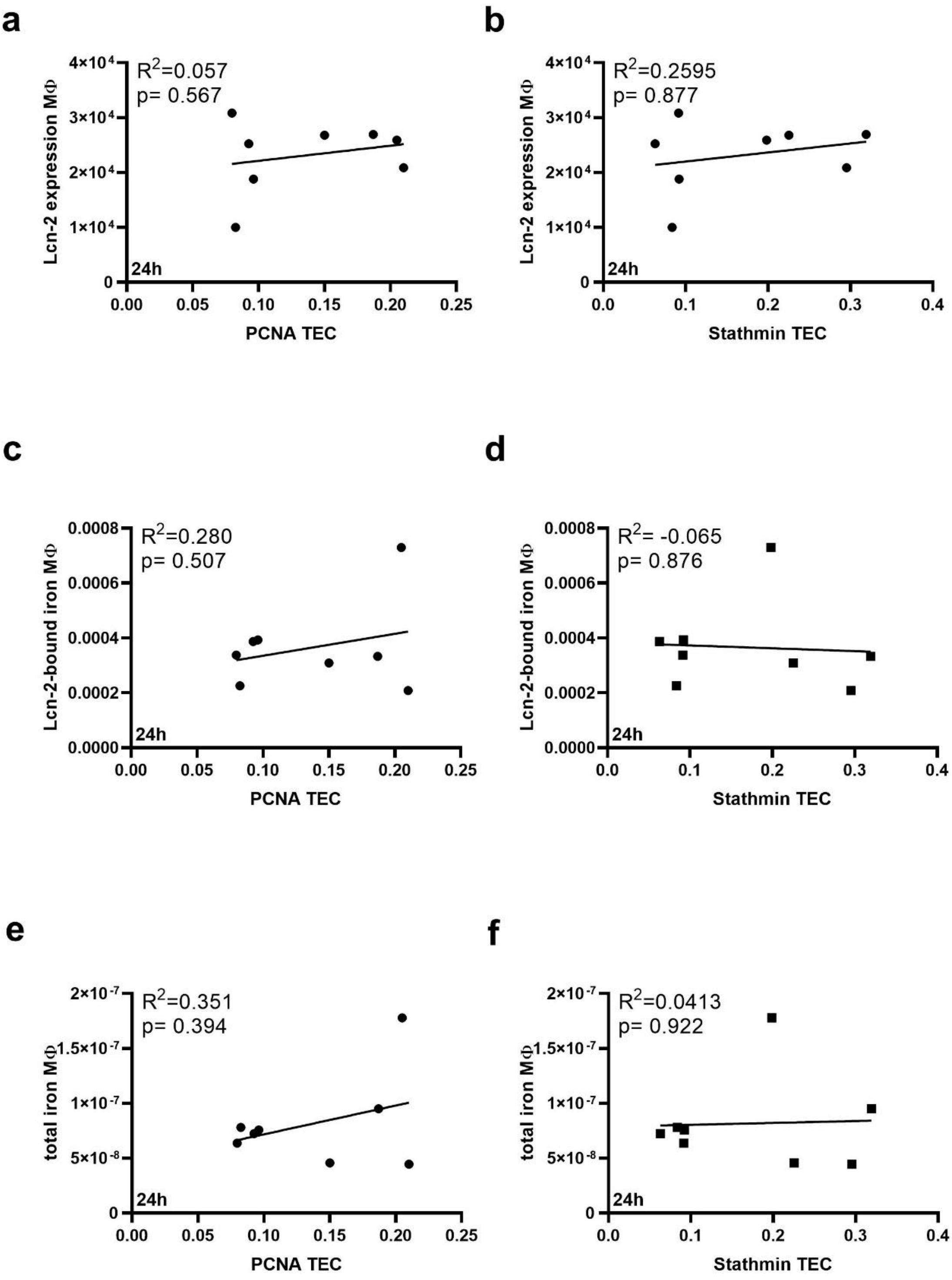

Supplemental Figure S3: Correlation to renal recovery markers at 24 h after CLP treatment

(a-f) Correlation between (a, c, e) PCNA or (b, d, f) Stathmin expression in TEC with either (a, b) Lcn-2 protein expression in renal MΦ, (c, d) Lcn-2-bound iron secreted from renal MΦ, or (e, f) total iron measured in the supernatant of renal MΦ (all values from the 24 h timepoint after CLP were included; R^2 and p-values are depicted in the individual graph).

Supplemental table S1: *Histological scoring.*

Kidney damage was assessed by evaluating histology via PAS staining. Poor (+), moderate (++), and severe (+++) scoring was applied and kidney sections were analyzed by screening for tubular detachment, tubular dilatation, tubular vacuoles, inflammatory infiltrate, and glomerular sclerosis. 5 independent mice were analyzed from Sham- and CLP-treated animals (24 h and 48 h).

**Sample**	**tubular detachment**	**tubular dilatation**	**tubular vacuoles**	**inflammatory infiltrate**	**glomerular sclerosis**
**Sham_1**	+	+	+	+	+
**Sham_2**	+	-	-	-	-
**Sham_3**	+	-	-	-	-
**Sham_4**	+	-	-	-	-
**Sham_5**	+	-	-	-	-
**CLP 24h_1**	++	++	-	++	++
**CLP 24h _2**	++	++	-	++	+++
**CLP 24h_3**	++	+	-	++	+++
**CLP 24h _4**	++	++	-	+++	+++
**CLP 24h _5**	+	++	-	++	++
**CLP 48h_1**	+	++	-	+	-
**CLP 48h _2**	+	++	-	+	-
**CLP 48h_3**	+	+	-	-	-
**CLP 48h _4**	+	+	-	-	-
**CLP 48h _5**	+	+	-	++	+
